# The Pathology of Acute Inflammation in Bone

**Published:** 1909-07-31

**Authors:** 


### SURGERY.

### THE PATHOLOGY OF ACUTE INFLAMMATION IN BONE.

The phenomena associated with the acute inflammation in bone are essentially the same as those of acute inflammation in other parts of the body: they are only modified by the anatomical structure of bone and the particular manner in which it reacts to injury or infection.

### Classification of Acute Bone Diseases.

We can divide acute bone disease into three classes—(*a*) traumatic, (*b*) infective, and (*c*) septic. By infective we mean that one of the pyogenic micro-organisms is responsible for the changes seen, but that it is brought to the affected region by the patient's blood stream. It is really an auto-infection, and for this reason the term auto-infective would be more exact. A septic inflammatory process, on the other hand, implies that the organisms have been introduced from without, as when osteomyelitis supervenes on an amputation, as the result of sepsis occurring at the time of operation.

It will thus be seen that the three classes are not divided by any hard and fast line, since an area of inflammation which is originally due to an injury may become invaded by septic organisms from the blood stream (infective) or from without (septic) if the original injury has happened to cause a wound in the superficial tissues covering the bone. As a matter of fact, a great number of the so-called infective inflammations are preceded by an injury which may be so slight as to pass almost unnoticed at the time. Acute epiphysitis occurring in infants is a typical example of this; and it is, therefore, not easy to put this affection in any one class.

If we regard the phenomena of inflammation as being divisible into the stage of reaction (vascular changes) and the stage of repair (connective-tissue cell changes), it will be found that the factors which are peculiar to bone inflammation belong to the latter class. The vascular changes consist, as in inflammation elsewhere, of a vaso-dilatation of the blood-vessels of the part with oscillation, slowing, and finally stasis of the blood stream, which is separated into a central layer of red blood corpuscles and a peripheral one of leucocytes. The whole object of this stage is to facilitate the outpouring of an exudate, whose function is to prepare the way for the processes of repair.

### Results of Acute Inflammation.

Acute inflammation in bone generally causes necrosis; this is due to interference with its blood supply. A long bone is supplied with blood from two sources, which are derived respectively from the nutrient artery and the periosteum. The former supplies the bone on its medullary aspect, while the latter consists of a number of small branches which enter the bone at right angles and nourish it superficially.

As the result of an injury to the surface of the bone, as, for instance, in the case of a blow on the subcutaneous portion of the tibia, a serous subperiosteal exudate is poured out and the periosteum is stripped up from the bone over the affected area. A flat area of bone is thus deprived of its blood supply, and undergoes necrosis. The dead bone so formed is known as a sequestrum; and in periostitis pure and simple it is flat and laminar in shape. The death of the bone may be prevented by early incision, or, on the other hand, infection may occur and the serous effusion then becomes a purulent one. The periosteum becomes thickened and inflamed, and the pus gradually makes its way to the surface, and a perforation is formed in the thickened periosteum which is known as a cloaca.

The dead bone underneath acts as a chronic irritant, and granulation tissue is deposited between the dead bone and the living, so that the sequestrum becomes separate and lies loose. Later, calcareous salts are deposited in the inflamed and hyperæmic periosteum, so that a new layer of false bone is formed. The ultimate fate of the sequestrum varies according to circumstances. It may be removed entire by surgical interference, or it may be broken up into a number of small pieces by the granulations and discharged piecemeal through the cloaca.

Whichever of these methods is employed, one thing is certain, and that is that healing will not take place until the entire sequestrum comes away or is removed.

### Osteo-myelitis.

So far mention has only been made of periostitis, but the features of the process are essentially the same, whatever part of the bone is attacked. Osteomyelitis is nearly always auto-infective The process starts generally in the delicate growing end of the diaphysis of a long bone where the medullary and periosteal blood supplies inosculate; either in a bone whose resistance has been diminished by a slight injury, or in an individual, generally a child, who is debilitated as the result of recent acute zymotic disease, such as measles or scarlet fever.

The infection causes thrombosis of the central vessels of the bone—*i.e.* the branches of the nutrient artery, and the sequestrum in this case, instead of being laminar, is tubular, involving the entire shaft. An abscess is formed in the medullary cavity. This most often burrows outwards along the epiphyseal line and becomes subperiosteal also; but in young children it may make its way straight through the epiphysis into the joint.

Since the periosteum is tightly bound down to the shaft of the bone at the epiphyseal line it follows that infection of the articulation from a subperiosteal abscess can only occur if the epiphyseal line lies inside the joint capsule. This is the case in the upper end of the ulna and the head of the femur. In other respects the pathology is the same as in periostitis. An involucrum forms all round the tubular sequestrum, and one or more cloacæ form, through which the sequestrum is gradually discharged in small portions.

